# Multisite survey of bacterial contamination in ready‐to‐eat meat products throughout the cooking and selling processes in urban supermarket, Nanjing, China

**DOI:** 10.1002/fsn3.1532

**Published:** 2020-03-24

**Authors:** Shao‐Kang Wang, Ling‐Meng Fu, Guo‐Wei Chen, Hong‐Mei Xiao, Da Pan, Ruo‐Fu Shi, Li‐Gang Yang, Gui‐Ju Sun

**Affiliations:** ^1^ Key Laboratory of Environmental Medicine and Engineering of Ministry of Education and Department of Nutrition and Food Hygiene School of Public Health Southeast University Nanjing China; ^2^ Environments and Bioengineering Institute Nanjing University of Science and Technology Nanjing China

**Keywords:** bacterial contamination, Chinese, pathogens, processing, RTE meat product, selling, supermarket

## Abstract

**Objective:**

Ready‐to‐eat (RTE) meat is a kind of popular instant food easily contaminated by microbes, which is one of the causes of foodborne diseases. This study analyzes the possible sources of RTE food bacterial contamination during processing and subsequent selling.

**Method:**

Samples of eight kinds of RTE meat were collected from four supermarkets in Nanjing, China. The knives, chopping boards, trays(containers of food), clamps, air, water, and hands of the sales staff were sampled, and the enumeration of aerobic plate count and total *coliforms* and *pathogenic bacteria* was performed.

**Results:**

The survey revealed that poor hygienic levels was the causes that RTE meat products were contaminated by bacteria at different levels. With regard to pathogen, the incidences of *Salmonella spp.* and *Staphylococcus aureus* were 4.2% and 2.1%, respectively. These results also revealed that the bacterial contamination of RTE food was caused by the air, as well as clamps, chopping boards, knives, trays, and hands of the operators. The total number of aerobic colonies were positively correlated with the amount of RTE food in one pot (*r* = .87728, *p* = .0217), and negatively correlated with the maximum temperature in the center of the meat (*r* = −.81633, *p* = .0475).

**Conclusion:**

The high number of bacteria in RTE foods indicates potential food safety risks and the need to improve the health of supermarket sales staff. The most important thing is to determine how to raise hygiene awareness of employees through food safety education. Meanwhile, a comprehensive set of regulations on hand cleaning and disinfection should be developed to facilitate public health and reduce foodborne illness caused by the consumption of RTE food.

## Graphical abstract

The hygiene condition of selling in supermarkets needs to be improved. The most important thing is to determine how to enhance the hygiene consciousness of employees through food safety education. Meanwhile, the government should formulate RTE food hygiene regulations and execute these in supermarkets.

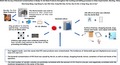

## 1 INTRODUCTION

Foodborne diseases is a growing public health problem worldwide and has brought considerable economic burdens to hospitals and other healthcare costs (Chen et al., 2019; Ghosh, Wahi, Kumar, & Ganguli, 2007). According to the reports of the United States Centers for Disease Control and Prevention (US‐CDC), the incidence of foodborne diseases in the United States is one in six cases, which results in 128,000 hospitalizations and 3,000 deaths each year. The Foodborne Disease Active Surveillance Network (Food Net) report stated that *Salmonella spp., Campylobacter spp., Shigella *spp., *Cryptosporidium *spp., and *Shiga toxin*‐producing *Escherichia coli *O157 continued to be the primary causes of the number and incidence of laboratory‐confirmed foodborne infections in the United States (Whittaker et al., 2009). Such food poisoning cases also often occur in China, in which approximately 500,000 illnesses and 12,000 deaths are reported every year (Zhou & Shen, 2010). Meanwhile, FERG report (Havelaar et al., 2015) suggests 1 in 10 people in the region where China is located develop foodborne diseases each year.

Ready‐to‐eat (RTE) food refers to the prepared food which can be consumed immediately or after taking a few steps such as heating before consuming (Microbiological Guidelines, 2007; Thienhirun & Chung, 2018). It is easily contaminated by a variety of foodborne pathogens and would be a major source of foodborne diseases. Meat and meat products are considered to be excellent sources of support for the growth of such pathogens (El‐Shenawy, Zaghloul, Abbass, & EsmailAI, 2016). As social, demographic, and consumption trends change, the proportion and types of meat and meat products, as well as RTE foods, have steadily increased on the international market (Havelaar et al., 2010; Sofos, 2008). Meanwhile, in recent years, RTE meat products have significantly risen in China on account of the increasingly urbanized lifestyle (Yang et al., 2016). The resulting foodborne diseases are also increasing (Sofos, 2008), so it caught our attention. From the farm to the consumer, the processing, transportation, and storage of meat products potentially provide growth conditions and nutrient contents to support microbial growth. Many sanitary surveys have revealed that RTE meat is a kind of instant food easily contaminated by microbes (Baloch et al., 2017; Yang et al., 2016). Since customers rarely reprocess these foods before consuming, food poisoning cases on account of RTE meat occasionally happen. Many studies have indicated the contamination of food caused by *Escherichia coli, Salmonella typhi, and Staphylococcus aureus *(*S. aureus*) during preparation, postcooking, and various handling stages (Ghosh, Wahi, Kumar, & Ganguli, 2007; Hanashiro, Morita, Matte, Matte, & Torres, 2005).

As for the item of business, the processing and selling of RTE meat products have been widely carried out in almost all large supermarkets at present. Hence, the sanitary condition of the processing and selling of RTE meat products was surveyed in several Chinese regions and other countries. GillespieI and Mitchell (2000) investigated cold sliced RTE meats from catering enterprises in the United Kingdom. They found that 74% of 3,494 samples met the standards of the European Union (EU), while 15 samples (<1%) were completely unacceptable. In Southern Italy supermarkets, 10% (105/1045) of selected RTE food samples were detected with *Listeria monocytogenes *(Di Pinto, Novello, Montemurro, & BonerbaE, 2010). In a word, unhygienic conditions behind RTE meat products may cause the final product to be contaminated with pathogens, which increases the risk of food poisoning in consumers.

Nanjing, located in eastern China, is the capital city of Jiangsu Province. It has a developed economy and a large consumption of RTE food. To understand hygienic condition and potential contamination sources throughout the RTE food processing and selling processes, the investigators organized this multisite survey to assay microorganisms of RTE food among the supermarkets in Nanjing, China.

## 2 MATERIALS AND METHODS

### 2.1 Targeted population and samples

Among the eight main urban areas of Nanjing, four urban areas were randomly selected, and a local supermarket was randomly selected in each urban area. These supermarkets belong to medium and large supermarkets and supply the food under investigation. Eight common RTE meat products were chosen, including marinated duck gizzards, saline chicken claws (chicken feet soaked in saltwater), soy‐sauced chicken wings, beef in brown sauce, sweet and sour spareribs, meatball, Braised pork, and pig knuckle in brown sauce.

### 2.2 Sample collection

The RTE food samples were all taken from processing and selling sites in selected supermarkets at a quantity of five times the test unit, stored in sterile containers, and sent to the laboratory for test within two hours. The sample test for bacterial status was required to be accomplished within 24 hr after sample collection.

Although the degree of contamination of different cooked food products through the processing and sales processes is slightly different, the overall change trend is the same, so the investigators chose the process of making and selling beef sauce as the representative, in the process of production and sale. Possible sources of contamination have been studied.

Raw materials, raw materials after splitting and shaping, and equipment in contact with beef in the main processes before hot processing, air, hands of operators, and water for production were sampled and analyzed. The sampling time was selected before the operation (preoperation), during the operation (in operation), and when the operation was completed (postoperation) of three consecutive days.

At the processing and production sites, samples of cooked food, semifinished products, cooked food products, and cooked food for 0, 2, and 4 hr were sampled. At the same time, it could sample the possible sources of microbial contamination in the process of food production, such as air, chopping boards, knives, the hands of workers, and clamps (a gripping device that is connected at both ends with pivot or hinged scissors for better handling of food for sale).

### 2.3 Determination of microbial load

#### 2.3.1 Homogenization step

A 25‐g edible part of RTE food was collected and placed into a sterile special bag containing 225 ml of sterile saline; subsequently, the sterile special bag was placed in the oscillation ring of the pulse homogenizer (Ningbo Scientz Biotechnology Co., LTD., China) to run homogeneously.

#### 2.3.2 Aerobic plate count

The samples were diluted into 10^2^–10^3^ dilution. One ml of liquid part was respectively poured into two sterile plates and mixed with the bacterial nutrient agar (NA) medium (HuanKai Microbial, China). Subsequently, the plates were incubated at 46°C ± 1°C for 48 hr. Then, plates with suitable amount of colonies were chosen to calculate the bacterial load ( Zhao et al., 2018).

### 2.4 Identification of *Coliforms, Salmonella *spp.,* Shigella *spp., and* S. aureus*

#### 2.4.1 Coliforms

*Coliforms* contamination test was carried out by most probable number (MPN) as described by Zhou and Jiao (2011). A 25‐g sample was placed in a sterile homogenization tube containing 225 ml of saline and tapped evenly with a tapping homogenizer to make a 1:10 sample homogenate. The sample was diluted serially in 10‐fold increments. For each sample, 3 suitable serial dilutions of sample homogenate were selected, 3 tubes of lauryl sulfate tryptone (LST, HuanKai Microbial, China) broth were inoculated at each dilution, 1 ml per tube (Double material LST broth was used for inoculation if the inoculum exceeded 1 ml), cultured at 36°C ± 1°C for 24 ± 2 hr, if air bubbles were observed in the inverted tube, 24 ± 2 hr gas production was taken for refermentation test, if no gas was produced, culture continued for 48 ± 2 hr, the gas‐producer was carried out a double‐fermentation test. The culture ring 1 was taken from the gas‐producing LST broth tube by inoculating loop, and transplanted into the tube of Brilliant Green Lactose Bile Salt Broth (BGLB, HuanKai Microbial), and cultured at 36°C ± 1°C for 48 ± 2 hr to observe if gas production happening. Gas generators were counted as coliform positive tubes. Based on the number of LST positive tubes in the coliform group, the MPN table was searched to report the MPN value of the coliform in each gram (ml) of the sample.

#### 2.4.2 *Salmonella *spp.

25 g of homogenized RTE sample was directly added into 225 ml of sterile saline. Then, 1 ml of the mixture was inoculated in 10 ml of minimal medium (MM) (Oxoid Limited, England) and selenite cystine (SC)(Oxoid Limited, England) medium, respectively. They were cultured at 42°C for 24 hr in MM, and at 36°C for 24 hr in SC. Subsequently, the cultures were streaked on bismuth sulfite (BS) and deoxycholate hydrogen sulfide lactose (DHL) agar plates, cultured at 36 ± 1°C for further observation and identification by biochemical analyses (Yue et al., 2014).

#### 2.4.3 *Shigella *spp.

25 g of homogenized sample was added into 225 ml of Gram‐negative (GN) (HuanKai Microbial, China) enrichment medium. After 6 hr of culture at 36 ± 1°C, the culture was streaked on hektoen enteric (HE) (HuanKai Microbial, China) and EMB (HuanKai Microbial, China) plates, respectively. The plates were incubated at 36 ± 1°C for 24 hr. Afterward, selected bacterial colonies were inoculated into triple sugar iron (TSI) agar and semisolid glucose, cultured at 36 ± 1°C for 24 hr. Morphology of bacteria was observed microscopically (Li, Chen, Wang, & Shao, 2008).

#### 2.4.4 S. aureus

25 g of sample was put into a sterile homogenous bag containing 225 ml of 7.5% sodium chloride broth and pat with a homogenizer, then cultured at 36 ± 1℃ for 18–24 hr. *S. aureus* showed turbid growth in 7.5% sodium chloride broth in severe contamination. The above culture crossed the vaccination, respectively, to Baird‐Parker Agar plate and blood agar plate at 36 ± 1℃for 18–24 hr. *S. aureus* grew on the Baird‐Parker Agar plate, the colony diameter was 2–3 mm, the color was gray to black, the edge was pale, surrounded by a turbid zone, and in its outer layer there was a transparent circle. The colony was contacted with the inoculation needle that has the hardness like cream to gum, and occasionally there were similar colonies that were not dissolved in fat, but no turbidity zone and transparent ring were observed. Colonies isolated from long‐preserved frozen or dried foods were less black than typical colonies and might appear rough and dry. On the blood agar plate, large colonies formed, round, smooth, raised, moist, golden yellow (sometimes white), completely transparent hemolytic circles could be seen around the colonies. The above colonies were selected for Gram staining microscopy and plasma coagulase test. Staining and microscopic examination would show that *S. aureus* was a *Gram‐positive coccus*, which was arranged in the form of grape ball, without spore cell and capsule, with the diameter of 0.5–1 m. If *S. aureus* could be observed under microscope, the result of plasma coagulase test was positive (Ling et al., 2013).

### 2.5 Measuring the internal temperature of the raw materials

The thermometer was used to measure the internal temperature of the raw materials. After taking the raw materials into the pan every 2–3 min, some of the cooked food was taken and cut, the thermometer mercury ball was wrapped to measure the internal temperature of the raw materials, and the maximum temperature reached and the time required to reach the maximum temperature (t_1_) were recorded. The internal temperature of the raw material was measured in the same manner every 4–5 min from the start of boiling of water, and it was recorded that it was maintained at 70°C or higher (t_2_).

### 2.6 Determination of the amount of microbes in air

Ordinary agar plates were scattered randomly at four corners and central on the floor of the processing and selling room, and an agar plate was placed in each 2–3 m^2^. After 15 min of exposure to air in the room, these plates were fully covered and sent to an incubator at 37°C. After 48 hr, potential bacterial colonies in each plate were counted (Yu, 2002).

### 2.7 Sampling from surface of knives, chopping boards, trays, clamps, and hands of the sales staff

Ten different parts (each, 50 cm^2^) of utensil and hands (hand back, palm, and fingertips) were wiped with sterile cotton swabs dipped in saline. A sterilized metal plate with a sterile stainless steel metal gauge hole of 5 cm^2^ (2.5 × 2.0 cm) was used to press the position of the selected point, and then a sterile cotton swab moistened with physiological saline was used to smear in the range of each plate hole. This operation was repeated 10 times. Then, the swabs were removed and placed in a triangular flask containing 50 ml of sterile saline with glass beads, which was shaken strongly to make the 1:1 sample liquid.

### 2.8 Sampling from water

To reduce the contamination during water sampling, the tap was flamed for 1–3 min. Then, the tap was burned with ignited cotton for 1–3 min. Afterward, the tap was turned to allow water to flow for 3–5 min prior to sampling. In the sampling process, 100–200 ml of water was collected into a sterile flask for further analysis (Zhang, Li, & Wu, 2015).

### 2.9 Statistical analysis

SAS software v9.0 (SAS Institute) was used for the statistical analysis. Pearson's correlation analysis was used to analyze the correlation coefficient between total bacterial count from each contamination source and total bacteria count measured in split beef. Pearson's correlation analysis was used to analyze the correlation coefficient between total bacterial count from each contamination source and total bacteria count measured in split cooked beef, the correlation coefficients between the heating conditions and the sterilization effect.

## 3 RESULTS

### 3.1 Survey of general health conditions

Each of the four supermarket community stores has a valid sanitary license and is equipped with special refrigerators or freezers. The area of the processing room is about 7–10 m^2^, the number of raw material cleaning pools is insufficient, and there is a phenomenon of mixing with the hand‐washing pool and cleaning in the same pool. There are no locker rooms in the four stores, and the workers' working clothes are in poor hygiene. Two of the four have no specialized raw material storage room, and the raw materials to be processed are randomly placed on the ground and the surface. The two stores with specialized raw material storage room have poor sanitary conditions, mainly due to the rotting of raw materials. It should be classified and stored off the ground and off the wall. There are hygienic dead corners on the floor and stove in the production room, and the facilities for preventing flies and rats are not perfect. All 30 employees randomly selected have health certificates.

### 3.2 Overall status of the bacterial contamination

A total of 96 food samples in eight categories were collected and tested according to the National Standard of the People's Republic of China (GB/ T4789.1–2008): Food hygiene microbiological inspection. As long as one of the indicators fails, the sample is evaluated as unqualified ( Ministry of Health of the People's Republic of China, 2008). The results revealed that 82.3% (79/96) of these samples had an acceptable count (the maximum allowable number is 80,000 CFU/g) of total aerobic bacteria, while 81.3% (78/96) of samples had an acceptable count (the maximum allowable number was 150 MPN/100 g) of total *coliforms* (Table 1). With regard to microbial pathogens, four of 96 samples were detected with *Salmonella spp*., while 2 of 96 samples were detected with *S. aureus*. However, *Shigella spp*. was not detected in any of the samples. The overall unqualified rate of sampled RTE meat was 31.3% (30/96; Table 1).

**Table 1:** Unqualified samples (%) of aerobic plate count, coliforms, and pathogen by RTE meat products

Product name	No. of samples	Aerobic plate count		Coliforms		Pathogen*	No. of Unqualified samples (%)
		CFU/g	Unqualified no. (%)	MNP/100g	Unqualified no. (%)	Positive no. (%)	
Marinated duck gizzards	12	9,900–83,000	3 (25.0)	60–270	1 (8.3)	1 (8.3)^a^	4 (33.3)
Saline chicken claws	12	20,000–88,000	2 (16.7)	≤30–270	2 (16.7)	1 (8.3)^a^	4 (33.3)
Soy‐sauced chicken wings	12	30,000–89,000	2 (16.7)	≤30–290	2 (16.7)	1 (8.3)^a^	3 (25.0)
Beef in brown sauce	12	4,700–83,000	2 (16.7)	≤30–190	2 (16.7)	1 (8.3)^b^	4 (33.3)
Sweet and sour spareribs	12	11,000–87,000	2 (16.7)	60–280	2 (16.7)	0	3 (25.0)
Braised pork	12	12,000–84,000	2 (16.7)	70–340	3 (25.0)	0	4 (33.3)
Meatball	12	18,900–85,000	2 (16.7)	90–420	4 (33.3)	1 (8.3)^a^	4 (33.3)
Pig knuckle in brown sauce	12	8,800–90,000	2 (16.7)	60–950	2 (16.7)	1 (8.3)^b^	4 (33.3)
Total	96	–	17 (17.7)	–	18 (18.8)	6 (6.3)	30 (31.3)

Note

Aerobic plate count >80,000 (CFU/g), enumeration of coliforms >150 (MPN/100g) and having pathogenic bacteria detected were regarded as disqualification.

Abbreviations: CFU, colony‐forming units; MNP, most probable number; RTE, ready‐to‐eat.

* Pathogen means *Salmonella *spp., *Shigella *spp., and *Staphylococcus aureus*;^a^ *Salmonella* spp.;^b^ *Staphylococcus aureus*.

### 3.3 Bacterial count during the production process

Raw materials, postsplit materials and utensils, air, the operator's hands, and water were sampled for three days. The sampling times were chosen at preoperation, in operation, and postoperation each day. After thawing, raw meat required further splitting for processing. Due to the different processing quantities, the segmentation processes took different periods of time. Three on‐site observations revealed that it usually took 30–50 min. As operation time went on, the number of bacteria in meat increased, regardless of the contamination source (Table 2). The present on‐site observations revealed that operators often used the same chopping board and knife to split different kinds of meat. In addition, it was noticed that the aerobic plate count of chopped beef was positively correlated with the total count of bacteria contained in the air, chopping board, knife, operator's hands, and post‐thawing beef (*p* < .05; Table 2).

**Table 2:** The bacterial counts from main contamination source according to different operation time before hot processing

Sampling time	From air per petri dish (aerobic plate count, CFU)	Smear experiment (aerobic plate count, CFU/cm^2^)				Water for production (/ml)		Post‐thawing beef (/g)		Split beef (/g)	
		Chopping board	Knife	Hand	Tray	Aerobic plate count (CFU)	Coliforms (MNP)	Aerobic plate count (CFU)	Coliforms (MNP)	Aerobic plate count (CFU)	Coliforms (MNP)
Preoperation	45	7.3 × 10^4^	6.5 × 10^2^	4.3 × 10^3^	4.5 × 10^3^	47	<30	3.1 × 10^5^	11,000	4.5 × 10^5^	≥24,000
In operation	93	9.6 × 10^4^	8.8 × 10^2^	3.8 × 10^4^	8.6 × 10^3^	56	<30	4.7 × 10^5^	≥24,000	8.6 × 10^5^	≥24,000
Postoperation	163	1.0 × 10^5^	1.4 × 10^3^	8.8 × 10^4^	2.6 × 10^4^	44	<30	5.4 × 10^5^	≥24,000	1.1 × 10^6^	≥24,000
Preoperation	30	5.1 × 10^4^	4.5 × 10^2^	5.2 × 10^3^	2.9 × 10^3^	100	<30	3.4 × 10^5^	≥24,000	5.7 × 10^5^	≥24,000
In operation	88	8.2 × 10^4^	7.6 × 10^2^	3.3 × 10^4^	6.4 × 10^3^	30	<30	5.8 × 10^5^	≥24,000	8.0 × 10^5^	≥24,000
Postoperation	132	9.8 × 10^4^	1.1 × 10^3^	6.9 × 10^4^	1.7 × 10^4^	26	<30	7.7 × 10^5^	≥24,000	2.1 × 10^6^	≥24,000
Preoperation	36	5.7 × 10^4^	4.9 × 10^2^	5.0 × 10^3^	3.7 × 10^3^	42	<30	4.0 × 10^5^	≥24,000	5.9 × 10^5^	≥24,000
In operation	95	8.7 × 10^4^	9.1 × 10^2^	3.9 × 10^4^	6.2 × 10^3^	80	<30	7.0 × 10^5^	≥24,000	8.3 × 10^5^	≥24,000
Postoperation	143	1.2 × 10^5^	1.5 × 10^3^	7.2 × 10^4^	1.5 × 10^4^	126	<30	9.9 × 10^5^	≥24,000	1.8 × 10^6^	≥24,000
Correlation coefficient^†^	*r* = .764	*r* = .756	*r* = .754	*r* = .788	*r* = .666	*r* = .092	–	*r* = .845	–	–	–
	*p* = .016	*p* = .018	*p* = .019	*p* = .012	*p* = .042	*p* = .814	–	*p* = .004	–	–	–

Note

† The coefficient and corresponding *p* value are calculated between total bacteria count from each contamination source and total bacteria count measured in split beef.

Preoperation (before the hot processing, in operation (during the hot processing), and postoperation (when the hot processing is completed).

Abbreviations: CFU, colony‐forming units; MNP, most probable number.

### 3.4 Bacterial contamination in the selling process

The results revealed that the bacterial contamination of RTE foods, chopping boards, knives, and food clamps increased with the time of selling. At the beginning of selling, chopping boards, knives, and food clamps were cleaned and dried, and it was found that the aerobic plate count was the lowest. After four hours of selling, the number of bacteria reached its peak value. It was also noticed that the aerobic plate count of split cooked beef was positively correlated with the aerobic plate count in the air, chopping board, knife, the operator's hands, and cook flap (*p* < .05, Table 3). The dynamic test on the hands of workers revealed that bacteria drastically increased after two hours of selling.

**Table 3:** The bacterial counts from main contamination source according to different selling time

Sampling time since selling onset	Air per petri dish (aerobic plate count, CFU)	Smear experiment (aerobic plate count, CFU/cm^2^)				Split cooked beef (/g)	
		Chopping board	Knife	Hand	Clamps	Aerobic plate count (CFU)	Coliforms (MNP)
0 hr	33	8.1 × 10^4^	4.3 × 10^2^	3.3 × 10^3^	3.5 × 10^2^	2.5 × 10^3^	70
2 hr	84	1.2 × 10^5^	5.5 × 10^2^	2.9 × 10^4^	4.1 × 10^2^	3.7 × 10^5^	110
4 hr	100	1.8 × 10^5^	9.7 × 10^2^	3.4 × 10^4^	5.1 × 10^2^	7.4 × 10^5^	150
0 hr	30	7.2 × 10^4^	3.8 × 10^2^	4.1 × 10^3^	2.9 × 10^2^	1.9 × 10^3^	60
2 hr	79	1.1 × 10^5^	4.9 × 10^2^	1.8 × 10^4^	3.4 × 10^2^	2.1 × 10^5^	90
4 hr	103	1.6 × 10^5^	8.1 × 10^2^	2.2 × 10^4^	4.7 × 10^2^	4.3 × 10^5^	110
0 hr	40	7.8 × 10^4^	3.6 × 10^2^	4.8 × 10^3^	3.4 × 10^2^	3.2 × 10^3^	<30
2 hr	94	1.6 × 10^5^	5.6 × 10^2^	2.0 × 10^4^	4.5 × 10^2^	5.4 × 10^5^	90
4 hr	132	2.0 × 10^5^	9.9 × 10^2^	2.4 × 10^4^	5.7 × 10^2^	8.1 × 10^5^	120
Correlation coefficient^†^	*r* = .787	*r* = .983	*r* = .922	*r* = .856	*r* = .959	–	–
	*p* = .012	*p* < .001	*p* = .004	*p* = .003	*p* < .001	–	–

Note

† The coefficient and corresponding *p* value are calculated between total bacterial count from each contamination source and total bacterial count measured in split cooked beef.

Abbreviations: CFU, colony‐forming units; MNP, most probable number.

### 3.5 Contamination source and bacterial load before thermal treatment

After first‐hand observations of the whole RTE food production process and communication with industrial professionals, it was well established that boiling is the main heating process for making RTE foods, which plays a decisive role in the residue of the microorganism. The sterilization for RTE food boiling in four supermarkets was assayed. The correlation test revealed that the sterilization effect (aerobic plate count at the beginning of heating minus those at the end of heating) was positively correlated with the quantity of meat in the pot (*r* = .880, *p* < .05), but was negatively correlated with the highest temperature in meat (*r* = −.817, *p* < .05) (Table 4).

**Table 4:** Heating versus microorganism residue on RTE foods

SN	Meat in the same pot(kg)	Highest temperature in meat (T, ℃)	Time to reach T (t_1_, min)	Time to maintain ≥70℃ (t_2_, min)	Aerobic plate count at the beginning and end of the heating(B/E, CFU/g)	Sterilization effect(B/E) CFU/g）
1	10	75	25	25	640,000/1800	638,200
2	8	78	25	21	560,000/890	559,110
3	15	71	30	22	1,200,000/1200	1,198,800
4	13	73	28	20	1,100,000/750	1,099,250
5	12	71	31	19	980,000/780	979,220
6	13	72	29	18	1,400,000/1300	1,398,700
Correlation coefficient^†^	*r* = .880	*r* = −.817	*r* = .762	*r* = −.623	–	–
	*p* = .021	*p* = .047	*p* = .078	*p* = .186	–	–

Note

† Correlation coefficients between the heating conditions and the sterilization effect.

Abbreviations: CFU, colony‐forming units; RTE, ready‐to‐eat; SN, serial number.

## 4 DISCUSSION

The present study aimed to analyze possible sources of RTE food bacterial contamination during its processing and subsequent selling in Nanjing. It was found that RTE meat samples were contaminated to different extents, and the occurrence of *coliform, Salmonella *spp., and *S. aureus* was determined in these products. Furthermore, there were various sources of bacterial contamination. As the operation time went on, the number of bacteria in meat increased, regardless of the source. In addition, the equipments used in the selling process and amount of microbes on the operators' hands directly put an impact on the hygienic condition of RTE food.

The overall unqualified rate of supermarkets RTE meat products was 31.3%. The aerobic plate count and coliforms were mainly exceeded, which is consistent with other reports in China (Guo, Hou, & Ge, 2007), Gu et al. have found unqualified rate of RTE meat products was 61.7% (29/47). The over‐standard rate of coliform bacteria was 61.7% (29/47). Among the 29 cooked meat products exceeding the national standard, the coliform group exceeded 100%; *Coliform bacteria* have been used as food safety and hygiene indicators in many countries because they can cause potential public health problems (Yang et al., 2018, Jay, Loessner, & Golden, 2005). *Coliform* specifically exists in warm‐blooded animals, which indicate that its presence in contaminated RTE meat products is mainly due to man‐made contamination and poor disinfection facilities available in the supermarket. At the same time, according to literature research, the detection rate of E. coli in RTE food exists all over the world (El‐Shenawy, Zaghloul, Abbass, Esmail, & Fouad, 2016; Gizaw, 2019). Compared with developed countries, there is more *E. coli* contamination in developing countries, which may be caused by poor temperature and air system management in food storage areas.

It is well known that different raw food materials are sensitive to specific bacteria, such as *S. aureus* to herbal food (Xing et al., 2014) and *Salmonella *spp. to chicken (De Oliveira et al., 2014). However, the raw materials or semiproducts might be contaminated by various kinds of bacteria, and even fungi and viruses. *S. aureus* contamination is important for food safety, since its virulence factors could cause illnesses with wide range of symptoms, ranging from diarrhea to bacteremia, resulting in high mortality rates (Sukhumungoon, Bunnueang, Butsabong, Sae‐Lim, & Rattanachuay, 2018). *S. aureus* is a pathogen which frequently causes community‐associated and nosocomial infections. It is also a major cause of foodborne disease worldwide due to its ability to produce different types of enterotoxins preformed in food (Kadariya, Smith, & Thapaliya, 2014). We have found 2 samples contaminated by *S. aureus*, which is one of the pathogens and is not allowed to appear in food. *S. aureus* is the species which is most often related to cases and outbreaks of food poisoning due to its ability to produce enterotoxins (Rodrigues, Gandra, Conceição, Silveira, & Timm, 2018). After *S. aureus* contaminating food, it can multiply at appropriate temperatures and produce staphylococcal enterotoxin (SE) and cause staphylococcal food poisoning (SFP) (Papadopoulos et al., 2019).

The aerobic plate count of split cooked beef was positively correlated with the aerobic plate count in the air, chopping board, knife, operator's hands, and clamps. This was correlated with studies conducted by Campos, Gil, Mourao, and PeixeL (2015) and Niyonzima et al. (2017), where kitchen utensils, food handlers, and work surfaces were identified as potential sources of bacterial contaminations in RTE foods. Keeratipibul, Techaruwichit, and Chaturongkasumrit (2009) have got that samples from processing lines which showed that cooked shrimps for sushi product were frequently contaminated during slitting step (3.85%). In addition, rowing (7.23%), sizing (6.93%), and peeling (6.27%) also contributed to coliforms contamination in the product. This demonstrates that the data of environmental swabs and in‐processed products contamination affecting the contamination of finished product can be correlated. Therefore, it was inferred that the equipments used in the selling process and amount of microbes on the operator's hands directly impact the hygienic condition of RTE food and was one of the main sources of contamination in the selling process. The contamination found in some utensils and equipments may evidence the inefficiency of cleaning and disinfection processes of tools and equipment that have direct contact with food (Rodrigues, Gandra, Conceição, Silveira, & Timm, 2018). The selling of RTE food also needs a very hygienic setting, and selling clerks are required to pay more attention to the hygiene of tools and their hands. Zhu et al. (2017) reported that only few people separated the cutting board between raw and RTE foods. The cross‐contamination of RTE foods from chicken meals via the cutting board, knife, and cooks’ hands increased the frequency of pathogen ingestion, and the risk of *salmonellosis*. Therefore, it is recommended to use different cutting boards for raw and cooked materials, and apply detergent and hot water during the cleaning procedure, in order to prevent the cross‐contamination of final products (Goh et al., 2014). Many researchers have indicated that wearing disinfected cloths, masks, and gloves, or washing hands with correct procedures using antibacterial soap or hand sanitizers could reduce bacterial cross‐contamination in food service environments (Bartz & Tondo, 2013; Montville, Chen, & Schaffner, 2002; Robinson et al., 2016). Wearing gloves can prevent food from being contaminated by *L. monocytogenes* and *Shigella* (Gallagher et al., 2016; Todd, Michaels, Greig, Smith, & Bartleson, 2010). Furthermore, food safety education should be provided and highlighted.

In addition to raw material issues, personal experience and other factors like hand hygiene also affect the residues of microbes (Lambrechts, Human, Doughari, & Lues, 2014). A number of residual microorganisms have been reported, since RTE foods were not heated or thoroughly cooked (Havelaar et al., 2010). Furthermore, although there are few unqualified RTE food products when these are taken away from the pot, as time goes on, microorganism contamination would inevitably occur in RTE food. Hence, focus is given on the process of postcooking of RTE food. The study conducted by Aung et al. (2016) revealed that the reheating by food handlers significantly reduced the overall median standard plate count (SPC) of food.

Even though the data in this study demonstrated the high bacterial number in RTE foods, suggesting the high risk of food safety concern, consumption of these foods from supermarkets is unavoidable. Therefore, it is suggested that food security training sessions for food operators should be organized to enhance their hygiene awareness. Meanwhile, a full set of regulations with regard to the cleaning and disinfecting of hands should be established for the benefits of public health.

## CONFLICT OF INTEREST

All authors have contributed significantly to the manuscript and declare that the work is original and has not been submitted or published elsewhere. None of the authors have any financial disclosure or conflict of interest.

## ETHICAL STATEMENT

Human and animal testing is unnecessary in our study.
